# New Agent for Detecting the Quantity of Urea in the Urine

**Published:** 1852-10

**Authors:** M. Liebig


Translated from the French.

*New Agent for Detecting the Quantity of Urea in the Urine.* By M. Liebig.

There are few questions in medicine more interesting than that which relates to the quantity of urea in the urine, because there are few diseases which do not modify more or less notably the proportion of this constituent. It will then be rendering an immense service to the art of curing, to provide physicians with a rapid and sure means of determining the proportion of this element.

Several valuable methods have already been discovered, but no one attains perfectly the end proposed. Some are faulty from their complication, others from their want of accuracy. One of the best consists in converting the urea into carbonate of ammonia and then determining, by known methods, the quantity of nitrogen; but it is evident that such a procedure requires a manipulation too long and too delicate to be conducted by physicians; it is, therefore, resorted to only in rare cases where rigorously exact analyses are desired.

Professor Liebig has discovered that urea combines with the binoxyde of mercury to form an insoluble compound, and he has founded upon this simple observation a method, which, without being perfectly exact, is at least very convenient for determining rapidly the proportion of urea in the urine.

He commences by preparing a solution of neutral nitrate of mercury in distilled water, so as to obtain a normal liquor, which is kept separately. Then when he wishes to examine the urine for the purpose of determining the quantity of urea which it contains, he adds gradually this normal liquor until the precipitate ceases. The quantity of the normal liquor added gives, within certain limits, a measure of the urea. Here, however, is a necessity for care. The precipitate which is formed is composed, as M. Liebig has shown, of 1 equivalent of urea
1 " of nitric acid,
1 " of the binoxyde of mercury.
From which it results that for each equivalent of urea precipitated in the new compound, there ought to be, and there is, in fact, three* equivalents of nitric acid which become free in the liquor. But the presence of this acid forms a serious obstacle to the further action of the nitrate, so that when the precipitate ceases to form, there is still a certain quantity of urea in the urine, which can only be thrown down after having saturated the free acid. This is done by adding gradually the water of baryta, taking care not to add it in excess, after which a new quantity of the normal liquor may be added, and a new precipitate obtained; and thus by successive additions of the normal liquor and the water of baryta, the whole of the urea may finally be precipitated. It is then that the whole quantity of normal liquor added furnishes a measure sufficiently exact of the quantity of urea.

This procedure has been repeated by Dr. Hoffman, who has spoken very favorably of it in a report which he has made to the Chemical Society of London, the 19th of January last. J.

* There seems here to be a mistake. According tothe general law of combination as many equivalents of acid are required to saturate a base as there are atoms of oxygen in the base. There would, therefore, be one equivalent of free nit. acid, instead of three as in the text.—[J.]

